# Attenuation of hedgehog/GLI signaling by NT1721 extends survival in pancreatic cancer

**DOI:** 10.1186/s13046-019-1445-z

**Published:** 2019-10-28

**Authors:** Claudia M. Kowolik, Min Lin, Jun Xie, Larry E. Overman, David A. Horne

**Affiliations:** 1 Department of Molecular Medicine, City of Hope National Medical Center, 1500 E. Duarte Road, Duarte, CA 91010 USA; 2 Department of Chemistry, 1102 Natural Sciences II, University of California, Irvine, CA 92697-2025 USA

**Keywords:** Pancreatic cancer, Epidithiodiketopiperazine, ETP, NT1721, GLI, Hedgehog signaling, Metastasis, Orthotopic model

## Abstract

**Background:**

Pancreatic cancer is one of the most lethal malignancies due to frequent late diagnosis, aggressive tumor growth and metastasis formation. Continuously raising incidence rates of pancreatic cancer and a lack of significant improvement in survival rates over the past 30 years highlight the need for new therapeutic agents. Thus, new therapeutic agents and strategies are urgently needed to improve the outcome for patients with pancreatic cancer. Here, we evaluated the anti-tumor activity of a new natural product-based epidithiodiketopiperazine, NT1721, against pancreatic cancer.

**Methods:**

We characterized the anticancer efficacy of NT1721 in multiple pancreatic cancer cell lines in vitro and in two orthotopic models. We also compared the effects of NT1721 to clinically used hedgehog inhibitors and the standard-of-care drug, gemcitabine. The effect of NT1721 on hedgehog/GLI signaling was assessed by determining the expression of GLI and GLI target genes both in vitro and in vivo*.*

**Results:**

NT1721 displayed IC_50_ values in the submicromolar range in multiple pancreatic cancer cell lines, while largely sparing normal pancreatic epithelial cells. NT1721 attenuated hedgehog/GLI signaling through downregulation of GLI1/2 transcription factors and their downstream target genes, which reduced cell proliferation and invasion in vitro and significantly decreased tumor growth and liver metastasis in two preclinical orthotopic mouse models of pancreatic cancer. Importantly, treatment with NT1721 significantly improved survival times of mice with pancreatic cancer compared to the standard-of-care drug, gemcitabine.

**Conclusions:**

Favorable therapeutics properties, i.e. 10-fold lower IC_50_ values than clinically used hedgehog inhibitors (vismodegib, erismodegib), a 90% reduction in liver metastasis and significantly better survival times compared to the standard-of-care drug, gemcitabine, provide a rational for testing NT1721 in the clinic either as a single agent or possibly in combination with gemcitabine or other therapeutic agents in PDAC patients overexpressing GLI1/2. This could potentially result in promising new treatment options for patients suffering from this devastating disease.

## Background

Pancreatic cancer is one of the most lethal cancers and the 4th leading cause of cancer-related deaths in the United States. Incidence rates have been on the raise over the past several years: According to the American Cancer Society an estimated 56,770 new cases will be diagnosed and 45,750 will die from the disease in 2019 [1]. Due to the lack of early symptoms and detection methods, most patients are diagnosed with pancreatic ductal adenocarcinoma (PDAC) at an advanced stage when the tumor has metastasized and is not resectable [2]. Gemcitabine became the standard-of-care drug for PDAC two decades ago, improving median survival compared to treatment with 5-fluorouracil (5.6 vs. 4.4 months) [3]. In recent clinical trials combinations of gemcitabine with other drugs resulted in relatively modest improvements of the median survival times [4]. A clinical trial with FOLFIRINOX vs. gemcitabine showed significantly improved median survival rates (11.1 vs. 6.8 months) in metastatic PDAC, but this regimen is associated with much higher toxicity rates that can only be tolerated by very few patients [5]. Despite these advances, overall prognosis and survival rates have not substantially improved over the past three decades, remaining at an overall 5-year survival rate of 1–5% for patients with metastatic PDAC [1, 2]. Major causes for treatment failure are aggressive growth, early metastasis, the development of gemcitabine resistance and enrichment for cancer stem cells (CSCs) that survive chemotherapy and promote tumor recurrence [2, 6, 7]. Hence, there is an urgent need to develop new drugs that can overcome the drug resistance and improve the outcome for patients with pancreatic cancer.

The sonic hedgehog (HH) pathway is a major regulator of cell proliferation, differentiation and polarity that is frequently aberrantly activated in a variety of cancers [4, 8, 9]. Canonical HH signaling is initiated through binding of sonic hedgehog (SHH) to its receptor PTCH1, which ultimately results in the activation of the zinc finger transcription factors GLI1/2 and the expression of GLI target genes [8]. GLI-dependent transcription can also occur in the absence of SHH since GLI transcription factors can be positively regulated by PI3K-AKT, KRAS or TGFβ1 [10–12]. GLI target genes (e.g. *GLI1, PTCH1, BCL2, BMI1, DNMT1, CCNE1, ABCG2*) are involved in a wide variety of cellular processes such as HH pathway feedback, proliferation, apoptosis and stem cell self-renewal [8, 13–16]. Aberrant activation of HH/GLI signaling has been implicated in increased proliferation, CSC signaling, epithelial-to-mesenchymal transition (EMT) and thus increased metastasis in several types of cancer including PDAC [16, 17]. Moreover, increased HH signaling may also contribute to gemcitabine resistance in PDAC. Gemcitabine-resistant PDAC cells display increased expression levels of members of the HH pathway, CSC and EMT markers compared to the gemcitabine-sensitive parental cells [7, 17]. Various studies have shown that blocking HH signaling with a SMO inhibitor (cyclopamine) decreases the IC_50_ values for gemcitabine, downregulates CSC markers and inhibits invasion and metastases in PDAC cells [17, 18]. Thus, targeting the HH/GLI pathway has emerged as promising strategy for the treatment of pancreatic cancer.

Epidithiodiketopiperazines (ETPs) are a broad class of fungal metabolites with potent antitumor activity in multiple solid and non-solid tumors. Here, we show for the first time that the biological activity of an ETP, NT1721, is associated with attenuated HH/GLI signaling. We demonstrate that NT1721 reduced the expression of GLI and GLI target genes in pancreatic cancer cell lines and two orthotopic mouse models. NT1721-mediated downregulation of genes associated with EMT resulted in significantly decreased metastases in vivo. Importantly, NT1721 also significantly increased survival times in an orthotopic model of pancreatic cancer compared to the control and gemcitabine-treated groups, highlighting its potential as new agent for the treatment of pancreatic cancer.

## Material and methods

### Reagents

NT1721 was synthesized as previously described [19]. Erismodegib and vismodegib were purchased from ApexBio (Houston, TX, USA).

### Cell culture

Panc1, Capan1, SU.86.86 and BxPC3 cells were obtained from ATCC (Manassas, VA, USA), authenticated by STR-profiling at the source and passaged for less than 6 months after receipt or resuscitation. The cells were cultured in DMEM (Panc1, Capan1) or RPMI (SU.86.86 and BxPC3) supplemented with 10% FBS (Atlas Biologicals, Fort Collins, CO, USA). Luciferase-expressing (luc^+^) Panc1 and Capan1 cells were generated as previously described [20]. Immortalized, non-tumorigenic human pancreatic duct epithelial cells (HPDEC) were obtained from AddexBio (San Diego, CA, USA). Normal primary human pancreatic epithelial cells were purchased from Cell Systems (Kirkland, WA, USA) and cultured in Complete Serum-Free Medium with RocketFuel™ (Cell Systems).

### Determination of IC_50_ values

The cell viability and IC_50_ values were determined as previously described [20] using the MTS assay (CellTiter 96® AQueous One Solution, Promega, Madison, WI, USA); briefly, 7500 cells/well were seeded in 96-well plates, cultured overnight and then treated with various concentrations of NT1721 for 48 h. Data from the MTS assay were expressed as percent of viable cells compared to the vehicle control (0.3% DMSO).

### Proliferation assay

Cells were stained with CSFE ((5(6)-Carboxyfluorescein N-hydroxysuccinimidylester, CellTrace™, ThermoFisher, Waltham, MA, USA) according to the manufacturer’s instructions, seeded at a concentration of 100,000 cells/well in 12-well plates and allowed to attach overnight. The cells were treated with NT1721 or DMSO the next day, harvested after 24, 48 or 72 h, stained with 0.2 μg/ml DAPI and subjected to FACS analysis. Fluorescence data were collected on a CyAN flow cytometer (Beckman Coulter, Brea, CA, USA) and analyzed with FlowJo software (TreeStar, Ashland, OR, USA).

### Cell cycle analysis

Cells were treated with NT1721 and harvested after 24 h, stained with propidium iodide (PI) (ThermoFisher) as previously described and subjected to FACS analysis [20].

### QPCR

Total RNA was isolated using the Direct-zol kit (Zymo Research, Irvine, CA, USA) and reverse transcribed using the Tetro cDNA synthesis kit (Bioline, Taunton, MA, USA).

The following qPCR primers were used:

- ACTB: 5’CCAACCGCGAGAAGATGA / 5’CCAGAGGCGTACAGGGATAG;
- ABCG2: 5’TGGCTTAGACTCAAGCACAGC / 5’TCGTCCCTGCTTAGACATCC;
- CCNE1: 5’GGCCAAAATCGACAGGAC / 5′ GGGTCTGCACAGACTGCAT;
- CDK2: 5′ AAAGCCAGAAACAAGTTGACG / 5’GTACTGGGCACACCCTCAGT;
- DNMT1: 5’CAAACCCCTTTCCAAACCTC / 5’TAATCCTGGGGCTAGGTGAA;
- GAPDH: 5’AGCCACATCGCTCAGACAC / 5’GCCCAATACGACCAAATCC;
- GLI1: 5’ACCCGGGGTCTCAAACTG / 5’GGCTGACAGTATAGGCAGAGC;
- GLI2: 5’CACGCTCTCCATGATCTCTG / 5’CCCCTCTCCTTAAGGTGCTC;
- PTCH1: 5’CATGTTTGCACCCGTCCT / 5’CCAGCACAGCAAGAAATACC;
- ZEB1: 5’CCTAAAAGAGCACTTAAGAATTCACAG / 5’CATTTCTTACTGCTTATGTGTGAGC;
- ZEB2: 5’AGGAGCTGTCTCGCCTTG / 5’GGCAAAAGCATCTGGAGTTC.

Quantitative PCR was performed using a CFX96 Touch Real-Time PCR detection system (Bio-Rad). Relative expression levels were calculated using the 2^−ΔΔCt^ method and GAPDH or ACTB as reference gene.

### Western blots

SDS-PAGE and Western blots were carried out as previously described [20]. Briefly, cells were lyzed using RIPA buffer (Cell signaling, Danvers, MA, USA) supplemented with Halt Protease inhibitor cocktail (ThermoFisher Scientific). The protein concentration was quantified using the Pierce BCA Protein Assay Kit (ThermoFisher Scientific). Equal amounts of protein were loaded on precast gels (Bio-Rad, Hercules, CA, USA), transferred to PVDF membranes (Bio-Rad) using the Trans-Blot Turbo Transfer System (Bio-Rad). Membranes were blocked for 1 h at RT in blocking buffer (5% w/v nonfat dry milk, 0.1% Tween-20 in TBS), incubated with primary antibodies overnight at 4 °C. Primary antibodies were purchased from Cell signaling (CDH2 (#13116), β-actin (#4970), BIM (#2933), BMI1 (#69640, DNMT1 (#5031), GAPDH (#5174), H3 (#4499), p21 (#2947), ABCG2 (#42078), BCL2 (#4223), CDK2 (#2546), MMP2 (#13132)), from Santa Cruz Biotchnology (GLI2 (sc-271,786), GLI1 (sc-20,687), ZEB2 (sc-271,984)), from Millipore (Burlington, MA, USA) (PTCH1 (06–1102)), from ThermoFisher (myc tag (# MA1–21316)) and from Genetex (Irvine, CA, USA) (ZEB1 (GTX55847)). The membranes were washed three times with TBS and then incubated 1 h at RT with the appropriate secondary antibodies. Bands were visualized using X-ray film or the ChemiDoc Imaging System (Bio-Rad) and analyzed with ImageJ or with Image Lab Software (Bio-Rad).

### GLI reporter assay

To establish a GLI reporter cell line, Capan1 cells were transduced with a lentiviral vector (pGreenFire1 tm-GLI-EF1-Neo, SBI System Biosciences, Palo Alto, CA, USA) that expresses firefly luciferase under the control of four GLI response elements. Transduced cells were selected with 600 μg/ml G418. Capan1 GLI reporter cells were seeded in 96-well plates (12,000 cells/well), allowed to attach over night and then treated with NT1721, erismodegib or vismodegib. The medium was removed after 36 h and D-luciferin (300 μg/ml) was added. The luminescence was measured after 5 min of incubation at 37 °C. The luminescence values were normalized to the protein concentration (Pierce BCA Protein Assay Kit) and then to the DMSO control.

### Invasion assay

To quantify the invasion of PDAC cell lines through the basement membrane we performed the CytoSelect™ 96-Well Cell Invasion Assay (Cell Biolabs, San Diego, CA, USA) according to the manufacturer’s instructions.

### GLI2 overexpression

Plasmid pCS2-MT GLI2 delta N (#17649) expressing GLI2ΔN, which displays strong transcriptional activity [21], was obtained from Addgene (Watertown, MA, USA). Panc1 and Capan1 cells were transfected with pCS2-MT GLI2 delta N or a GFP-expressing control plasmid using jetPrime transfection reagent (Polyplus New York, NY, USA) according to the manufacturer’s instructions. The cells were trypsinized after 16 h, seeded into multiple wells, allowed to attach (8 h) and treated with NT1721 for 48 h.

### In vivo studies

Mouse care and experimental procedures were performed under pathogen-free conditions in accordance with approved protocols from the institutional animal care and use committee of City of Hope National Medical Center. For the orthotopic model, 5 × 10^5^ luciferase-expressing Panc1 or Capan1 cells were injected into the pancreas of 6-to 8-week old male NSG mice (Jackson Laboratory, Bar Harbor, ME, USA). To determine the tumor burden, we injected the mice (I.P.) with 3 mg D-Luciferin (Promega) 12 days after tumor injection and imaged them in an IVIS 100 (Caliper Life Sciences). A standard region of interest (ROI), which included the entire mouse, was used to determine the total body bioluminescence. Data were expressed as photons/s/mm^2^. The mice were then distributed into groups bearing equal tumor burdens and treated with NT1721 (30 mg/kg on 3 consecutive days per week by gavage) or gemcitabine (100 mg/kg, twice per week on non-consecutive days by I.P. injection). The control groups for NT1721 and gemcitabine received the vehicle control (5% DMSO / 30% solutol (Sigma, St. Louis, MO, USA)) and PBS, respectively.

### Microscopy

H&E (Hematoxylin & Eosin) stained tissue slides were prepared from formalin-fixed mouse livers using standard methods. Pictures of these tissue slides were taken using a Zeiss Observer Z1 Widefield Microscope with a 506 Axioam Color Camera (Tiling option in Zen Blue software, 10% overlap) and analyzed with Image Pro Premier software (Media Cybernetics) to determine the number of tumor foci/μm^2^.

### Statistical analysis

The mean ± standard deviation (SD) was calculated for each treatment group. Unless noted otherwise, the 2-tailed t-test with Welch’s correction was used to determine statistical significance between two treatment groups. The log-rank test was used to evaluate the statistical significance in survival curves. *p* values < 0.05 were considered to be significant.

## Results

### NT1721 displayed IC_50_ values in the nanomolar range in multiple PDAC cell lines

**Fig. 1:**
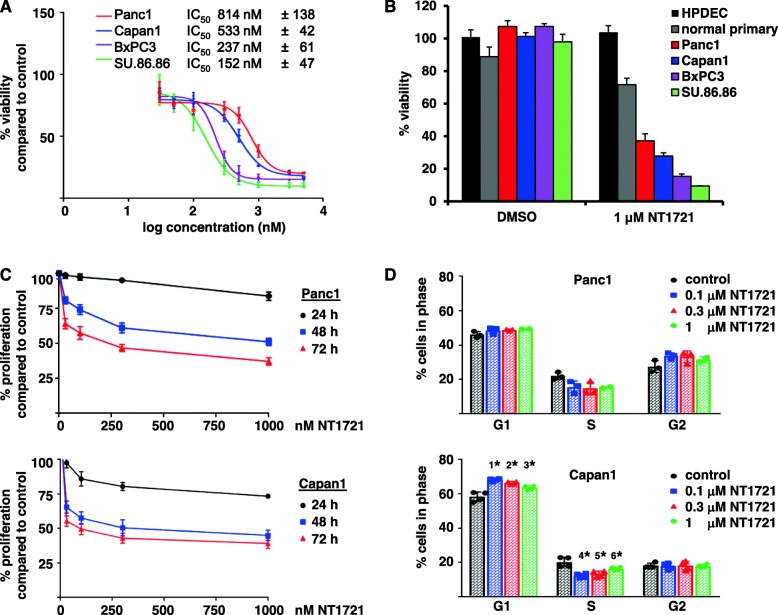
IC_50_ values of NT1721 and its effects on proliferation and cell cycle in PDAC cells. **a** Pancreatic cancer cell lines were treated with increasing concentrations of NT1721. The cell viability and IC_50_ values were determined after 48 h. The IC_50_ values represent the mean ± SD from three experiments. **b** Effect on normal pancreatic epithelial cells. PDAC cell lines, normal primary pancreatic epithelial cells and immortalized, non-tumorigenic pancreatic duct epithelial cells (HPDEC) were treated with 0.3% DMSO or NT1721 and the cell viability was determined after 48 h using untreated cells as control. **c** Proliferation inhibition. PDAC cells were stained with CSFE, treated with NT1721 and subjected to FACS analysis to determine the mean fluorescence intensity after 24, 48 or 72 h. The data were normalized to the control. The values represent the mean ± SD from three experiments. **d** Cell cycle analysis. The cells were treated with NT1721, stained with PI after 24 h and analyzed by FACS. The symbols (1* - 6*) indicate statistically significant differences compared to the control with *p* values of 0.0005, 0.0015, 0.015, 0.0022, 0.0054 and 0.0388, respectively. The values represent the mean ± SD from four experiments

To evaluate the potency of NT1721 against PDAC we treated four pancreatic cancer cell lines (derived from either primary tumors (Panc1, BxPC3) or from liver metastases (Capan-1, SU.86.86)) with NT1721 and determined their viability and the IC_50_ values after 48 h. As shown in Fig. 1a, the IC_50_ values were all < 1 μM, ranging from ~ 150 to 800 nM, depending on the cell line. To assess the effect of NT1721 on normal cells we also treated normal primary as well as immortalized, non-tumorigenic pancreatic duct epithelial cells (HPDEC) with NT1721. As shown in Fig. 1b, NT1721 decreased the viability of the PDAC cell lines while ≥75% of the normal primary and HPDEC cells remained viable at a concentration of 1 μM NT1721, suggesting that NT1721 may preferentially decrease the viability of pancreatic tumor cells. We used invasive Panc1 and Capan1 cells, expressing mutated KRAS, which is prevalent in the majority of PDAC patients, to investigate the effect of NT1721 on PDAC in vitro and in vivo*.*

### NT1721 decreased proliferation and cell cycle progression

We investigated the effect of NT1721 on the proliferation rate by staining Panc1 and Capan1 cells with CSFE and treating them with NT1721 for 24 to 72 h. FACS analysis of live cells showed that relatively low concentrations of NT1721 (30 nM) significantly decreased proliferation in both Panc1 and Capan1 cells after 48 h by 19 and 48%, respectively (*p* = 0.0003 for both). Treatment with 30 nM NT1721 for 24 h led only in Capan1 cells to significantly reduced proliferation (*p* = 0.001), but not in Panc1 cells (Fig. 1c). We then assessed the effect of NT1721 on the cell cycle by staining NT1721-treated cells with PI after 24 h. NT1721 significantly increased the percentage of cells in G1 phase and decreased the S phase in Capan1 cells compared to the control (Fig. 1d), which is in line with the decreased proliferation we detected in this cell line after 24 h. The result suggests that NT1721 decreased cell cycle progression in Capan1 cells after 24 h. In contrast, Panc1 cells did not display a significant change in the cell cycle after 24 h, which is not surprising given that we did not observe a change in proliferation at this early time point (Fig. 1c,d).

### NT1721 downregulated GLI transcription factors and their downstream target genes

**Fig. 2:**
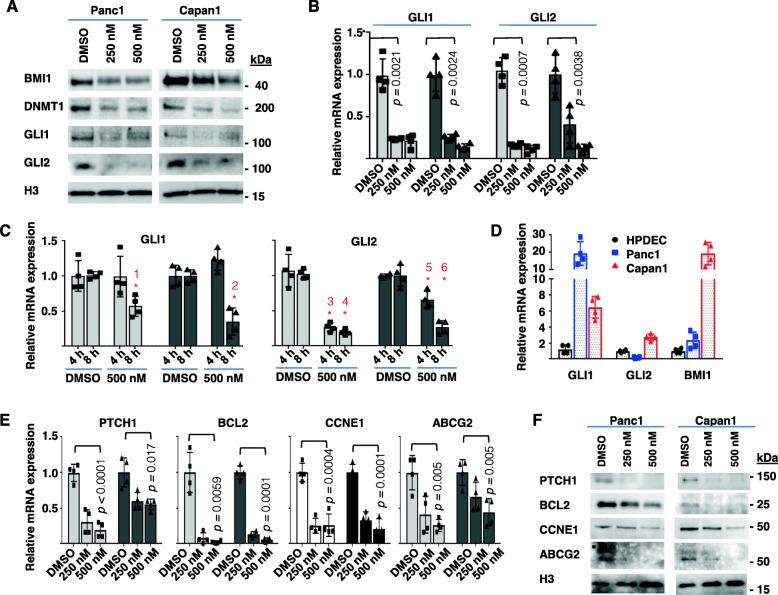
NT1721-mediated downregulation of GLI and GLI target genes. **a** PDAC cells were treated with NT1721 and protein expression levels were assessed by Western blot after 36 h. **b-e** The qPCR data were analyzed using GAPDH as reference gene. The data represent the mean ± SD from 4 independent experiments. **b** GLI1 and GLI2 mRNA levels were determined in Panc1  and Capan1  cells after treatment with NT1721 for 24 h. **c** Comparison of GLI1, GLI2 and BMI1 mRNA levels in PDAC and non-tumorigenic HPDEC cells. **d** NT1721-mediated time-dependent downregulation of GLI mRNA in Panc1  and Capan1  cells. The asterisks indicate statistically significant differences compared to the respective controls: 1* *p =* 0.0055*;* 2* *p =* 0.0032*;* 3* *p =* 0.0045*;* 4* *p <* 0.0001*;* 5* *p =* 0.0083*;* 6* *p =* 0.0004. **e** mRNA downregulation of GLI target genes (PTCH1, BCL2, CCNE1, ABCG2) in Panc1  and Capan1  cells after 24 h treatment with NT1721. **f** Western blots of GLI target genes (PTCH1, BCL2, CCNE1, ABCG2)

Since we have previously shown that NT1721 decreased DNMT1 and BMI1 expression in AML cells [20] we first confirmed on the protein level that NT1721 also downregulated these genes in PDAC cells (Fig. 2a). Given that both genes are targets of GLI transcription factors [13, 22] we investigated whether their downregulation might be (at least partially) mediated by decreased GLI signaling: RNAseq data from various cancer cell lines suggested that treatment with NT1721 led to the downregulation of GLI1 and GLI2, which we then confirmed on the mRNA (Fig. 2b) and protein level (Fig. 2a) in Panc1 and Capan1 cells. A time-course experiment revealed that NT1721 (500 nM) significantly decreased GLI1 and GLI2 mRNA levels after 8 h of treatment. However, GLI2 mRNA expression was significantly reduced earlier (after 4 h) and to a much greater degree in both cell lines compared to GLI1 expression (Fig. 2c), suggesting that GLI2 may be a major (direct or indirect) target of NT1721. To confirm that GLI signaling was aberrantly activated in Panc1 and Capan1 cells we compared GLI mRNA expression levels of PDAC and non-tumorigenic HPDEC cells: GLI1 mRNA levels were overexpressed in both PDAC cell lines by 6–20-fold. GLI2 mRNA levels were only elevated in Capan1 cells (Fig. 2d). These results confirmed the aberrant activation of the HH pathway in these cell lines since high GLI1 mRNA expression levels are considered to be a reliable indicator of activated HH signaling; high GLI2 levels are also a sign of activated HH signaling because GLI2 induces GLI1 transcription by binding to its promoter [23]. In contrast, upregulation of GLI2 by GLI1 is presumed to be indirect [24]. Moreover, BMI1 was also significantly overexpressed in both PDAC cell lines compared to HPDEC cells (Fig. 2d), which is in line with reports showing that BMI1 expression depends on HH pathway activity in several tumors [16, 25, 26]. To further verify that GLI-mediated signaling was attenuated after treatment with NT1721 we determined the expression levels of additional downstream targets of the GLI transcription factors: PTCH1, BCL2, CCNE1 and ABCG2 [9, 15, 26, 27]. QPCR analyses revealed that these genes were also downregulated after treatment with NT1721 for 24 h (Fig. 2e). Downregulation of PTCH1, BCL2, CCNE1 and ABCG2 was also confirmed on the protein level (Fig. 2f). Taken together, these results suggest that NT1721 may decrease HH/GLI signaling through downregulation of GLI transcription factors.

### NT1721 displayed significantly lower IC_50_ values than clinically used hedgehog pathway inhibitors

**Fig. 3:**
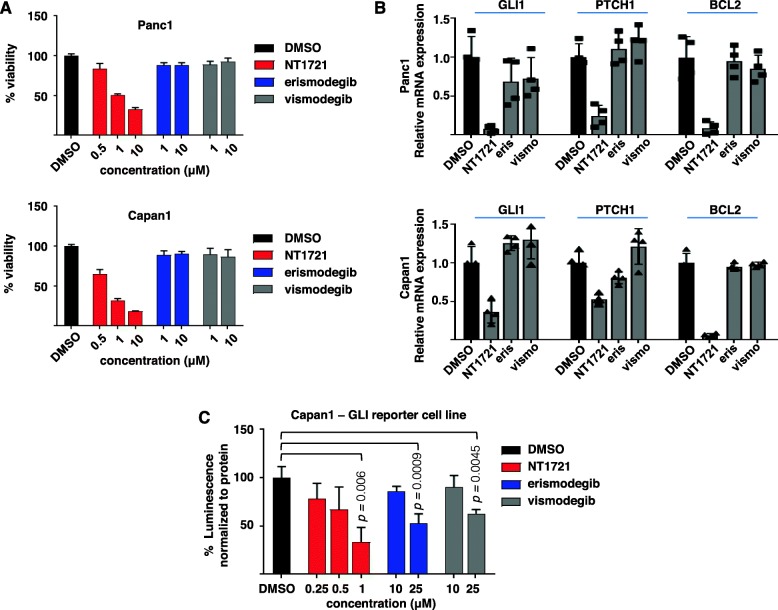
NT1721 reduced PDAC cell viability and hedgehog/GLI signaling significantly better than clinically used hedgehog inhibitors. **a** Viability of Panc1 and Capan1 cells treated with NT1721, erismodegib or vismodegib. **b** PTCH1 and BCL2 mRNA expression in and Capan1 cells treated with NT1721, erismodegib or vismodegib. **c** Capan1 GLI reporter cells were treated with NT1721, erismodegib or vismodegib. The bioluminescence was measured after 48 h and normalized to the control. The graphs represent the mean ± SD from triplicate values

To compare the efficacy of NT1721 and clinically used hedgehog inhibitors (i.e. erismodegib, vismodegib) against PDAC we treated Panc1 and Capan1 cells with the drugs or NT1721. Treatment of Panc1 cells for 48 h with 1 or 10 μM NT1721 reduced the viability by 50 and 69%, respectively, while treatment with 10 μM erismodegib or vismodegib resulted only in a 7–12% reduction in viability (Fig. 3a). Our results are in line with previous reports showing that vismodegib did not significantly reduce Panc1 viability at low micromolar concentrations (1 μM) [28–30]. Similar effects were observed in Capan1 cells: Treatment of Capan1 cells with 1 or 10 μM NT1721 reduced the viability by 69 and 82%, respectively, while treatment with 10 μM erismodegib or vismodegib reduced the viability only by 10–13%. These results indicate that 10-fold lower concentrations of NT1721 led to a significantly better reduction in cell viability in both Panc1 and Capan1 cells compared to erismodegib and vismodegib. To assess the effect of the drugs on hedgehog signaling we also compared their effect on the expression of hedgehog/GLI target genes. Treating PDAC cells with 10 μM erismodegib or vismodegib did not significantly reduce the GLI1, PTCH1 and BCL2 mRNA expression (Fig. 3b). In contrast, treatment of PDAC cells with 500 nM NT1721 was sufficient to significantly decrease the expression of GLI1, PTCH1 and BCL2, demonstrating that NT1721 reduced the expression of hedgehog/GLI target genes at 20-fold lower concentrations compared to erismodegib and vismodegib. To further assess the effect of the drugs on hedgehog/GLI signaling, we established a Capan1 GLI reporter cell line using a lentiviral vector that expressed luciferase under the control of four GLI response elements. We treated the Capan1 GLI reporter cell line with the drugs and measured the luciferase activity after 36 h. Treatment with 1 μM NT1721 was sufficient to significantly reduce the luminescence while 10-fold higher concentrations of erismodegib or vismodegib did not significantly affect the luminescence (Fig. 3c). However, a significant reduction in luminescence was observed after treatment with 25 μM erismodegib or vismodegib, indicating that NT1721 decreased the luminescence and thus hedgehog/GLI signaling at 25-fold lower concentrations. Taken together, our data show that NT1721 reduced hedgehog/GLI signaling at significantly lower concentrations in both Panc1 and Capan1 cells compared to erismodegib and vismodegib.

### NT1721 induced the expression of tumor suppressor genes and decreased the invasive potential of PDAC

**Fig. 4:**
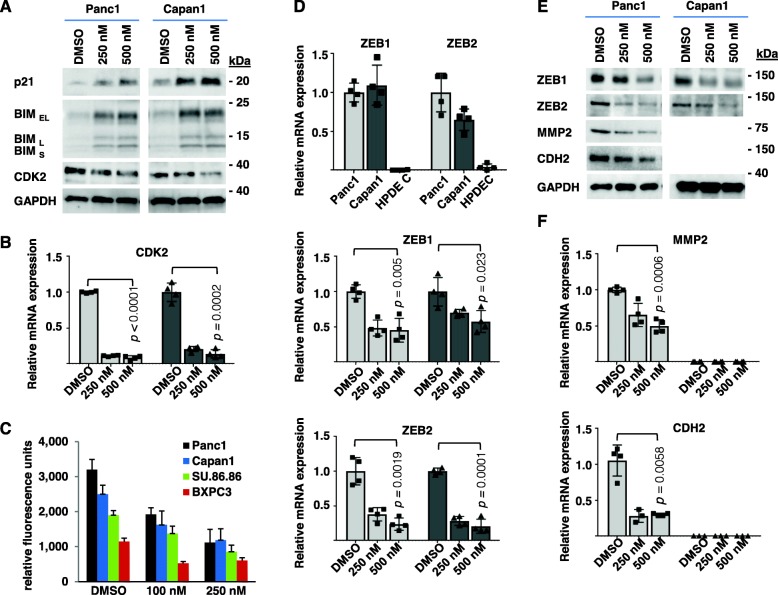
NT1721 induced tumor suppressor genes and decreased the invasive potential of pancreatic cancer cell lines. **a** NT1721-mediated induction of tumor suppressor genes (BIM, p21) and CDK2 was assessed by Western blot after 24 h using GAPDH as loading control. **b** CDK2 mRNA expression was determined after 24 h treatment of Panc1  and Capan1  cells with NT1721; GAPDH was used as reference gene. The graphs represent the mean ± SD from 4 independent experiments. **c** PDAC cell lines were treated with NT1721 for 16 h. Their invasive potential was quantified using the CytoSelect™ 96-Well Cell Invasion Assay (Cell Biolabs). **d** qPCR to determine ZEB1 and ZEB2 mRNA expression levels in PDAC cells and non-tumorigenic HPDEC cells. GAPDH was used as reference gene. The graphs represent the mean ± SD from 4 experiments. **e** Protein expression of EMT markers (ZEB1, ZEB2, CDH2, MMP2) was determined by Western blot in Panc1  and Capan1  cells after 48 h treatment with NT1721. **f** The mRNA expression of EMT markers (MMP2, CDH2) was assessed in Panc1  and Capan1  cells after 24 h treatment with NT1721

Decreased BMI1 expression has been linked to the re-expression of several tumor suppressor genes including BIM (BCL2L11), p21 (CDKN1A) [31, 32]. Thus we examined whether the NT1721-mediated reduction of BMI1 expression induced the re-expression of these tumor suppressors. As shown in Fig. 4a, NT1721 led to a strong upregulation of BIM and p21 in both cell lines. Treatment with NT1721 was also associated with a significant, > 80% decrease in CDK2 mRNA levels in both cell lines (Fig. 4b), which was confirmed on the protein level (Fig. 4a). This is in line with previous reports showing that lower BMI1 expression levels are associated with decreased CDK2 expression and thus cell cycle progression and proliferation [32–34].

Since aberrant HH/GLI signaling is linked to increased EMT, invasion and metastasis in various cancers including PDAC [16, 17] we assessed the effect of NT1721-mediated GLI downregulation on the invasive potential of PDAC cells: We used the CytoSelect™ Cell Invasion Assay (Cell Biolabs) to quantify the ability of NT1721-treated PDAC cell lines to migrate through the basement membrane. NT1721 significantly reduced the migration through the basement membrane in a concentration-dependent manner by 2–3-fold in all PDAC cell lines (Fig. 4c). To investigate the changes leading to the decreased invasion we then examined the expression of several genes associated with EMT and invasion: Previous reports show that HH/GLI signaling can indirectly induce the EMT-associated expression of ZEB1 and ZEB2 [27, 35]. Thus, we first verified that the ZEB transcription factors were upregulated in the PDAC cells by comparing the mRNA expression levels in Panc1, Capan1 and non-tumorigenic HPDEC cells: Both ZEB1 and ZEB2 were highly expressed in Panc1 and Capan1 cells compared to HPDEC (Fig. 4d), suggesting that GLI signaling may (at least partially) drive their expression. Treatment of PDAC cells with NT1721 reduced the mRNA expression of both ZEB1 and ZEB2 in Panc1 and Capan1 cells (Fig. 4d) and downregulation of ZEB1 was also confirmed on the protein level (Fig. 4e). We then also investigated whether NT1721 (similar to HH inhibitors such as GANT61, erismodegib and cyclopamine) could decrease the expression of CDH2 and MMP2 [36–38]. As shown in Fig. 4f, NT1721 decreased the mRNA expression of both CDH2 and MMP2 in Panc1 cells. Downregulation of CDH2 was also confirmed on the protein level in Panc1 cells (Fig. 4e). No CDH2 mRNA or protein expression was detected in Capan1 cells (Fig. 4e,f). Taken together, the data suggest that NT1721 reduced the invasive potential of PDAC cells through downregulation of multiple EMT markers.

### Antitumor effects of NT1721 depend at least partially on GLI downregulation

To investigate the importance of GLI downregulation for the antitumor effects of NT1721 we overexpressed myc-tagged GLI2ΔN [21] in PDAC cells (Additional file 1: Figure S1a). We compared the effect of NT1721 treatment on the viability of GLI2ΔN-overexpressing and GFP-expressing control cells. As shown in Additional file 1: Figure S1b, treatment with a low concentration of NT1721 (250 nM) failed to reduce the viability of GLI2ΔN-overexpressing cells in both cell lines while significantly reducing the viability of GFP-expressing cells by 20–30%. Treatment with 500 nM NT1721 did not reduce the viability of NT1721-treated GLI2Δ2-expressing Capan1 cells while reducing the viability of the control cells by 50%. The viability of Panc1 cells treated with 500 nM NT1721 was reduced to a lesser extend in GLI2Δ2-expressing cells compared to the control cells. These results suggest that the effects of NT1721 may at least partially relay on GLI downregulation. To further investigate this we then determined the effects of NT1721 on GLI1, BCL2 and ZEB1 mRNA levels in GLI2ΔN-overexpressing and GFP-expressing control cells: As shown in Additional file 1: Figure S1c, GLI2ΔN overexpression prevented GLI1 downregulation in both Panc1 and Capan1 cells after treatment with 250 nM NT1721 while the GLI1 mRNA levels were reduced by > 80% in NT1721-treated, GFP-expressing control cells. This result was expected given that GLI2 induces GLI1 transcription [23]. NT1721 failed to downregulate BCL2, a direct GLI target gene, in GLI2ΔN-expressing cells while NT1721 reducing BCL2 expression levels by > 70% in control cells (Additional file 1: Figure S1c). We also assessed ZEB1 expression in these cells since forced GLI2 expression has been shown to increase ZEB1 expression in pancreatic cancer cells [39]. As shown in Additional file 1: Figure S1c, ZEB1 was downregulated to a significantly lesser degree in GLI2ΔN-overexpressing cells compared to GFP-expressing control cells. Taken together, these results suggest that the antitumor effects of NT1721 may at least partially depend on GLI downregulation.

### NT1721 reduced tumor growth, metastasis and significantly prolonged survival in orthotopic mouse models of pancreatic cancer

**Fig. 5:**
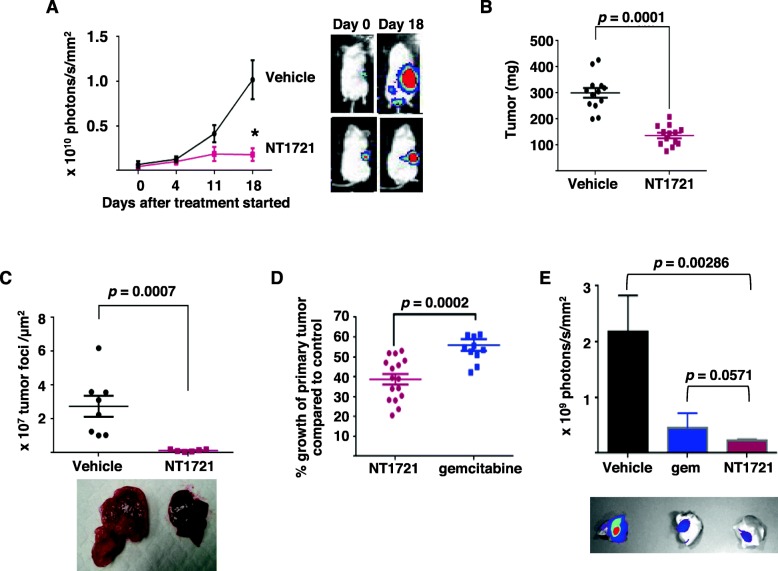
NT1721 inhibited tumor growth and reduced liver metastases in an orthotopic model of pancreatic cancer. Male NSG mice bearing orthotopically growing Panc1 luc^+^ tumors were treated 12 days after tumor injection with 30 mg/kg NT1721 (three times per week), gemcitabine (100 mg/kg, twice per week) or with the vehicle control. **a** Bioluminescent signals. The mice were imaged on the indicated days (*n* = 9). * indicates a significant difference between groups on day 18 (*p =* 1 × 10^− 6^). Representative pictures of mice from both groups are shown on the right. **b** Tumor weight after 5 weeks of treatment with NT1721. **c** Tumor foci in the liver (*n* = 8). Tissue slides of livers from mice treated with either NT1721 or gemcitabine for 5 weeks were prepared and the number of tumor foci was quantified as described in the Material and methods section. Representative pictures of whole livers from control and NT1721-treated mice are shown below the graphs. **d** Comparison of the in vivo efficacy of NT1721 and gemcitabine. The graph shows the percentage of tumor growth in NT1721- and gemcitabine-treated mice compared to the average in control mice (data from 2 independent experiments). **e** Decreased metastasis formation in liver. The mice (*n* = 4 per group) were injected with D-luciferin after 5 weeks of treatment with NT1721 or gemcitabine and then euthanized. The livers were immediately harvested and used for bioluminescent imaging. The Mann-Whitney test was used to determine the *p* values indicated above the graphs

To study the in vivo efficacy of NT1721 against pancreatic cancer we first treated NSG mice bearing orthotopically growing Panc1 luciferase-expressing (luc^+^) tumors with 30 mg/kg of NT1721 for 3 consecutive days per week or the vehicle control (5% DMSO / 30% solutol in PBS) [20]. Bioluminescent imaging revealed a statistically significant ~ 4 fold difference between the treatment and the control groups 18 days after the treatment started (*p =* 1 × 10^− 6^) (Fig. 5a). The mice were euthanized after 5 weeks of treatment and the weight of the primary tumor was determined. As shown in Fig. 5b, the tumor weight was significantly lower (by 54%) in treated mice compared to the control mice (*p =* 0.0001). To investigate the effect of NT1721 on liver metastasis we harvested the livers after 5 weeks of treatment, prepared tissue slides and quantified the number of tumor foci/μm^2^ in the liver tissue from NT1721-treated- and control mice. As shown in Fig. 5c, NT1721 significantly reduced the number of tumor foci in the liver (by 92%) compared to control mice (*n* = 8, *p =* 0.0007), indicating that NT1721 efficiently reduced metastasis formation in the liver. We then compared the antitumor efficacy of NT1721 and gemcitabine by treating tumor-bearing mice with NT1721 (30 mg/kg) or gemcitabine (100 mg/kg, twice per week) (*n* = 16). As shown in Fig. 5d, we detected a significant difference in tumor growth between NT1721- and gemcitabine-treated mice: Compared with the control mice, tumor growth was reduced by 61% in the NT1721-treated mice and by 44% in the gemcitabine-treated group (*p =* 0.0002), suggesting that NT1721 may inhibit tumor growth better than gemcitabine. To assess the influence of NT1721 and gemcitabine on metastasis we treated mice bearing luc^+^-tumors for 5 weeks, injected D-luciferin, euthanized them and immediately compared the bioluminescent signals from the livers in the control and treatment groups (*n* = 4). Livers from the NT1721-treated group displayed 8-fold lower bioluminescent signals than the control group; in contrast, livers from the gemcitabine-treated mice showed only a 5-fold reduction in bioluminescent signals compared to the control (Fig. 5e). The difference between the control and the NT1721 group was statistically significant (*p =* 0.00286). However, the difference between the NT1721- and gemcitabine-treated groups did not reach statistical significance (*p =* 0.0571).

**Fig. 6:**
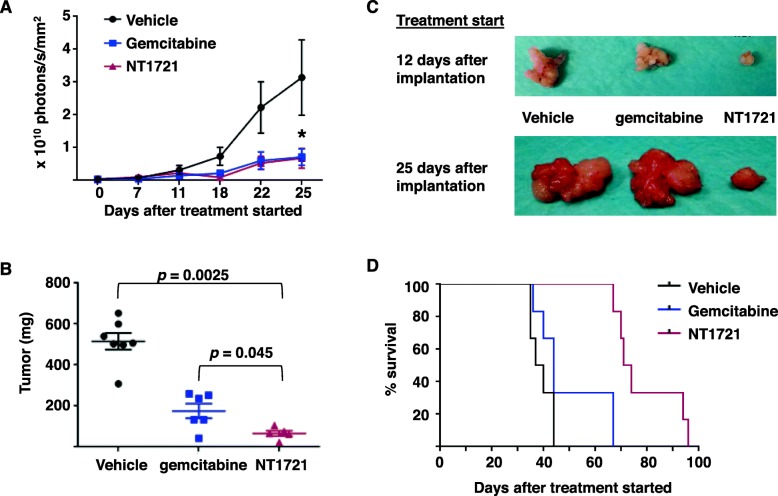
NT1721 displays better antitumor efficacy than gemcitabine and prolongs survival of mice with advanced pancreatic cancer. Male NSG mice were orthotopically implanted with Capan1 luc^+^ cells. Treatment started with 30 mg/kg NT1721 (three times per week), gemcitabine (100 mg/kg, twice per week) or the vehicle control (*n* = 7 per group) 12 days (**a**, **b**) or 25 days (**d**) after cell implantation. **a** Bioluminescent signals. The mice were imaged on the indicated days. The asterisk indicates a significant difference between the control group and treatment groups (NT1721 or gemcitabine) on day 25 (*p* < 0.05). **b** Tumor weight after 5 weeks of treatment with NT1721 or gemcitabine. *P* values were determined using Mann-Whitney test and are indicated in the graphs. **c** Photos of the representative primary tumors from mice that were treated with NT1721 or gemcitabine and euthanized after 5 weeks of treatment. **d** Survival curves. Treatment with NT1721 or gemcitabine started on day 25 after tumor implantation (*n* = 6). The log-rank test was used to determine *p* values: The difference in survival between the control and gemcitabine treated group was significant (*p =* 0.029); the difference between the gemcitabine-treated and the NT1721-treated group was also significant (*p =* 0.0007)

We then compared the efficacy of NT1721 and gemcitabine in a second mouse model: NSG mice bearing orthotopically growing Capan1 luc^+^ tumors were treated with NT1721, gemcitabine or the vehicle control. Bioluminescent imaging showed that both NT1721 and gemcitabine reduced the tumor growth in treated mice compared to the control group in a comparable manner during the first 25 days of treatment (Fig. 6a). However, weighing the primary tumors after 5 weeks of treatment revealed that mice treated with NT1721 had smaller tumors compared to mice treated with gemcitabine. NT1721-treated mice showed an 82% reduction in tumor growth while gemcitabine decreased the tumor growth only by 66% compared to the control group. The difference in tumor weight between the NT1721- and the gemcitabine-treated group was statistically significant (*p =* 0.045) (Fig. 6b,c). To compare the influence of NT1721 and gemcitabine on tumor growth and survival times of mice bearing more advanced tumors Capan1 luc^+^ tumors were allowed to grow orthotopically for 25 days (instead of only 12 days) before the treatment started: NT1721 clearly decreased the tumor growth of the more advanced tumors while gemcitabine did not cause an obvious decrease in tumor growth (Fig. 6c). As shown in Fig. 6d, treatment with NT1721 statistically significantly prolonged the median survival time to 71 days compared to gemcitabine-treated mice (44 days, *p =* 0.0007) and the control group (39 days, *p =* 0.0007). Taken together, these results show that NT1721 might be more efficacious against advanced tumors than gemcitabine.

### NT1721 attenuated HH/GLI signaling in vivo

**Fig. 7:**
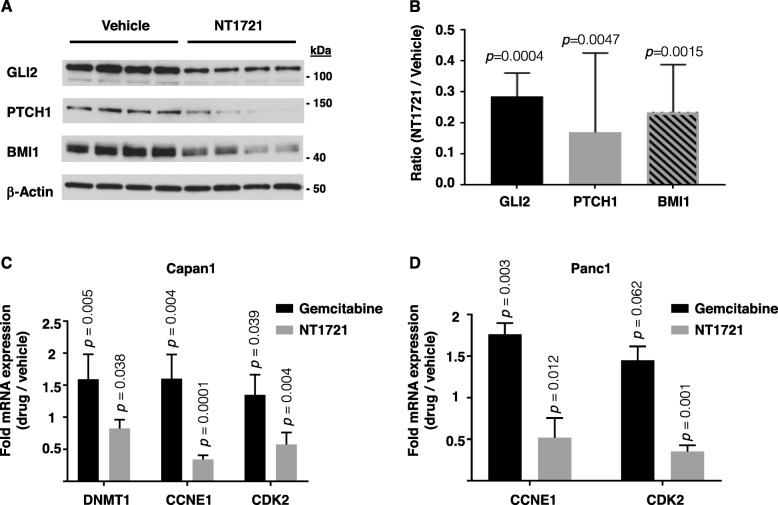
NT1721-mediated changes in gene expression in vivo. **a** Tumors from individual mice bearing Capan1 tumors were harvested and used for Western blots analysis (*n* = 4 per group). **b** Band intensities from (**a**) were quantified with ImageJ software and normalized to β-actin expression. The graphs represent the ratio of mean value of treated mice to control mice ± SD. *p* values for the comparison of treated and control mice are indicated above the graphs. **c** and **d** Tumors from mice bearing Capan1 tumors (**c**) or Panc1 tumors (**d**) were harvested after 5 weeks of treatment with NT1721 or gemcitabine and used for qPCR analysis (*n* = 4 per group). The data were normalized to ACTB. The graphs show the fold difference ± SD in mRNA expression in NT1721- or gemcitabine-treated mice compared to control mice. *P values* for the comparison of treated and control mice are indicated above the graphs

To assess the effect of NT1721 on GLI signaling in vivo we used tumor tissue from individual mice bearing Capan1 tumors for Western blot and qPCR analysis of GLI2 and GLI target genes. As shown in Fig. 7a,b, treatment with NT1721 reduced the protein expression of GLI2, PTCH1 and BMI1, statistically significantly by 72, 83 and 78%, respectively, recapitulating the decreases seen in PDAC cell lines in vitro. We also compared the effect of gemcitabine and NT1721 on the mRNA expression of *DNMT1*, a direct target gene of GLI1 [13]. *DNMT1* expression was significantly reduced in NT1721-treated mice, while gemcitabine-treated mice showed *DNMT1* upregulation (Fig. 7c). Taken together, our data suggest that NT1721 attenuated GLI signaling in vivo.

NT1721 also significantly reduced the mRNA expression of two genes involved in cell cycle progression, *CCNE1* and *CDK2*, by 64 and 45%, respectively, in Capan1 tumors (Fig. 7c) and by 50 and 60%, respectively, in Panc1 tumors (Fig. 7d). The results indicate that NT1721 might, at least partially, decrease the cell cycle progression and thus tumor growth through CCNE1 and CDK2 downregulation. In contrast, CCNE1 and CDK2 mRNA expression in gemcitabine-treated mice was significantly upregulated in both Capan1 and Panc1 tumors compared to the control group. Since upregulated expression of CDK2, CCNE, DNMT1 is associated with accelerated cell cycle progression and thus tumor growth [33, 40] these results suggest that the tumors might have started to develop a drug resistance after initially responding to the treatment with gemcitabine (Fig. 6a,c).

## Discussion

PDAC is still one of the deadliest cancers since 80% of patients have unresectable, metastatic disease at the time of diagnosis and more than 80% of patients undergoing surgery relapse [4]. New treatment options are desperately needed since the outcome for PDAC patients has not significantly improved over the past three decades.

Here we investigated the anti-PDAC activity of a new ETP, NT1721. The biological activity of ETPs has been linked to various molecular mechanisms [41–43]. We show for the first time that an ETP, NT1721, can attenuate HH signaling through downregulation of the main mediators of HH signaling, GLI1 and GLI2. Treatment of PDAC cells with NT1721 decreased the expression of GLI target genes associated with EMT/ metastasis, drug resistance and the self-renewal of CSCs. These results are in line with several reports showing that HH inhibition can reduce CSC growth, chemoresistance and EMT in PDAC [17, 18, 35, 44, 45]. It is noteworthy that several of the genes (GLI1, BMI1, DNMT1, ZEB1, ZEB2) that were downregulated by NT1721 have prognostic value in PDAC since high expression levels of these genes are associated with shorter survival in PDAC patients [40, 46–48]. Reducing GLI expression has also the potential to decrease treatment failure due to an acquired drug resistance: Several studies have demonstrated that treatment with gemcitabine induced GLI expression, which led to GLI-mediated upregulation of stem cell genes such as CD24, ABCG2, BMI1 and gemcitabine resistance [7, 44, 49]. Resistance to a BRAF inhibitor, vemurafenib, has also been linked to GLI overexpression [36]. Moreover, PDAC cells displaying an acquired resistance to the BET bromodomain inhibitor JQ1 showed dramatically elevated GLI2 levels [50]. Treatment of these resistant cells with HH inhibitors or GLI knockdown restored their drug sensitivity [7, 36, 44, 49, 50]. Taken together these studies indicate that inhibiting the HH pathway could improve the prognosis for PDAC patients by potentially preventing treatment failure due to inherent or acquired upregulation of tumor stem cell and/or drug resistance genes.

HH inhibitors such as cyclopamine (SMO inhibitor) or GANT61 (GLI inhibitor) have shown promising results in vitro and in vivo against PDAC: Cyclopamine decreased the viability of several PDAC cell lines, reduced invasion and reversed gemcitabine resistance [17, 18, 51]. However, cyclopamine, like other SMO-targeting hedgehog inhibitors (erismodegib, vismodegib), had no effect on HH ligand-independent cell lines such as Panc1 at low micromolar concentrations (< 10 μM) ([17, 51], Fig. 3). A report by Yauch et al. shows that the sensitity of PDAC cell lines to SMO-based HH inhibitors does not correlate with GLI1 or SMO mRNA expression levels at low drug concentrations; the authors concluded that the decrease in cell viability at high concentrations of SMO inhibitors may be due to off-target effects [52]. However, GANT61, which directly targets GLI downstream of SMO, inhibited the growth of HH ligand-independent Panc1 cells with an reported IC_50_ value of 5 μM after 48 h treatment [53]. Thus, GLI inhibitors like GANT61 are advantageous in cancers where SMO contains an activating mutation or GLI activation is SHH ligand-independent and driven by e.g. KRAS mutations or by TGFβ-driven GLI2 expression [12]. Since NT1721 potently (IC_50_ < 1 μM) inhibited the growth of HH ligand-independent Panc1 cells it is unlikely that NT1721 targets SMO or the HH pathway upstream of SMO. Given that GLI2 can directly induce GLI1 transcription and given that NT1721 downregulated GLI2 mRNA expression at an earlier time point and to a greater degree than GLI1 mRNA expression it seems possible that NT1721 interfered with TGFβ-induced GLI2 mRNA expression. However, the exact mechanism how NT1721 mediates GLI downregulation needs to be further investigated.

## Conclusion

In summary, NT1721 displayed better efficacy in controlling tumor growth and metastasis in preclinical orthotopic mouse models of PDAC than the standard-of-care drug, gemcitabine. Importantly, NT1721 is orally available, well tolerated in mice and significantly prolonged survival compared to the standard-of-care drug, gemcitabine. Thus, our data provide a rational for testing NT1721 in the clinic either as a single agent or potentially in combination with gemcitabine or other therapeutic agents in PDAC patients overexpressing GLI transcription factors.

## Supplementary information

**Additional file 1: Figure S1.** Antitumor effects of NT1721 depend at least partially on GLI downregulation. **a** Overexpression of myc-tagged, activated GLI2 (GLI2Δ2) in Panc1 and Capan1 cells. **b** Viability of GLI2ΔN-overexpressing and GFP-expressing control cells after treatment with 250 nM NT1721. The symbols (1* - 3*) indicate statistically significant differences compared to the control with *p* values of 0.003, 0.01 and < 0.001, respectively. **c** Expression levels of GLI1, BCL2 and ZEB1 in GLI2ΔN-overexpressing and GFP-expressing control cells after treatment with 250 nM NT1721. The graphs represent the mean ± SD from triplicate values.

## Data Availability

All data generated or analyzed during this study are included within the article.

## Abbreviations

CSCs: Cancer stem cells
CSFE: 5(6)-Carboxyfluorescein N-hydroxysuccinimidylester
EMT: Epithelial-to-mesenchymal transition
ETP: Epidithiodiketopiperazine
HH: Hedgehog
HPDEC: Human pancreatic ductal epithelial cells
MFI: Median fluorescence intensity
PDAC: Pancreatic ductal epithelial carcinoma
