# Supplementary figures and images for: Attenuation of hedgehog/GLI signaling by NT1721 extends survival in pancreatic cancer
Source: J Exp Clin Cancer Res. 2019 Oct 28;38:431. doi: 10.1186/s13046-019-1445-z (PMC6819529; doi:10.1186/s13046-019-1445-z)

A

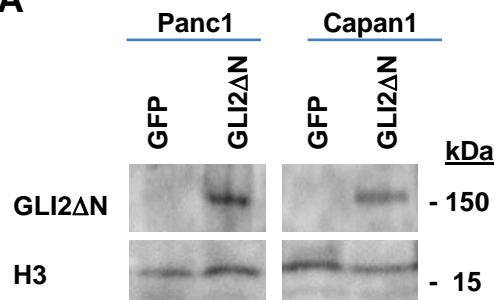

B

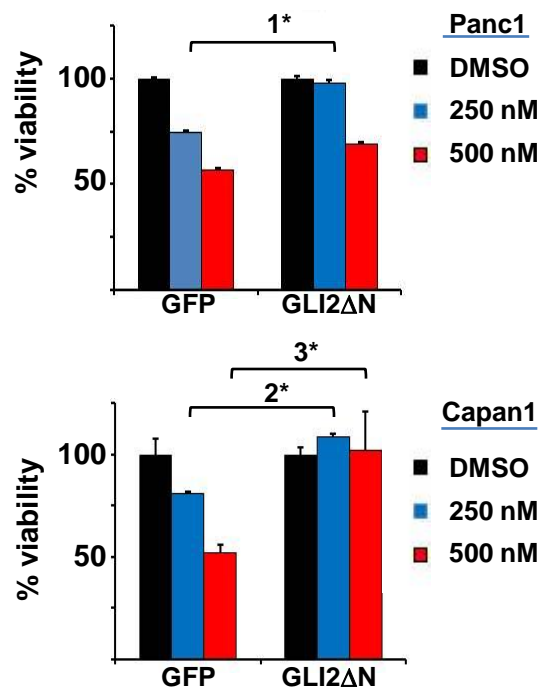

C

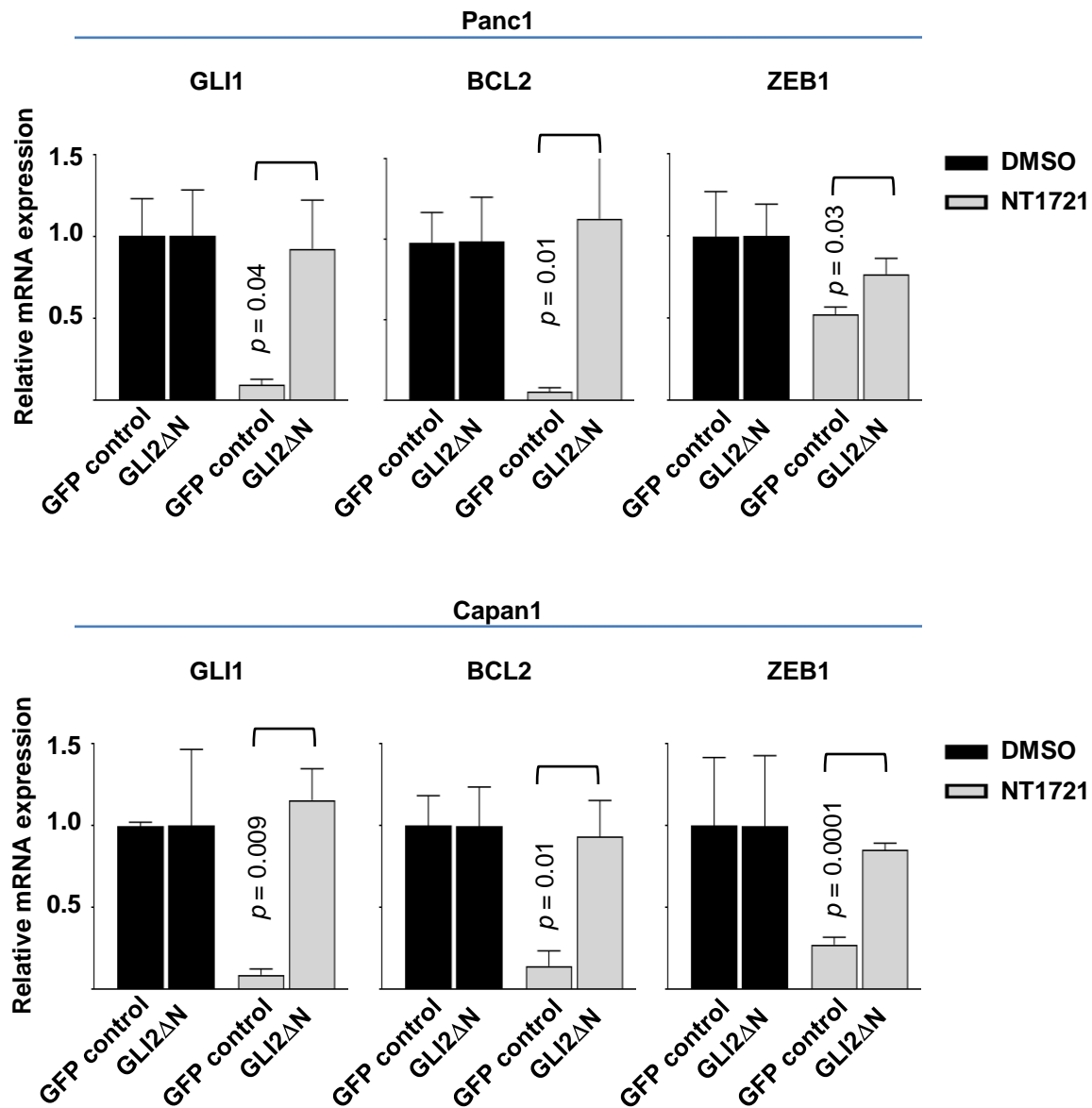

Supplement: Supplementary file 1 — Additional file 1: Figure S1. Antitumor effects of NT1721 depend at least partially on GLI downregulation. a Overexpression of myc-tagged, activated GLI2 (GLI2Δ2) in Panc1 and Capan1 cells. b Viability of GLI2ΔN-overexpressing and GFP-expressing control cells after treatment with 250 nM NT1721. The symbols (1* - 3*) indicate statistically significant differences compared to the control with p values of 0.003, 0.01 and < 0.001, respectively. c Expression levels of GLI1, BCL2 and ZEB1 in GLI2ΔN-overexpressing and GFP-expressing control cells after treatment with 250 nM NT1721. The graphs represent the mean ± SD from triplicate values. [file 13046_2019_1445_MOESM1_ESM.pdf]
